# Independent prognostic value of left atrial function by two-dimensional speckle tracking imaging in patients with non -ST-segment-elevation acute myocardial infarction

**DOI:** 10.1186/s12872-015-0135-9

**Published:** 2015-11-04

**Authors:** Chunlai Shao, Jing Zhu, Jianchang Chen, Weiting Xu

**Affiliations:** Department of Cardiology, The Second Affiliated Hospital of Soochow University, Sanxiang Street 1055, Suzhou, China

**Keywords:** Two-dimensional speckle tracking imaging, Strain, Strain rate, Left atrial function, Non-ST-segment-elevation acute myocardial infarction

## Abstract

**Background:**

The objective of this study is to evaluate left atrial(LA) function and its prognostic value by two-dimensional speckle tracking echocardiography (STE) in patients with non-ST-segment-elevation acute myocardial infarction (NSTEAMI).

**Methods:**

Global longitudinal LA S/SR data obtained by 2D speckle imaging with automated software (Echo PAC, GE Medical).

**Results:**

Clinical variables and angiographic, echocardiographic, and STE parameters were studied in 65 patients with NSTEAMI (51 males and 14 females; mean age of 60.7 ± 9.8 years) who underwent elective PCI. The final study population consisted of 51 individuals (43 males and 8 females; mean age of 62.9 ± 11.1 years) and a 12 ± 3 months follow-up was performed. A total of 22 combined cardiovascular events(20 patients) occurred. With the use of Univariable Cox regression, all parameters were evaluated in the prediction of cardiac events, ischemic events, and/or cardiac death. According to ROC analysis, baseline mean global left atrial SRs (ROC area 0.82, *p* = 0.001) and baseline mean global left atrial SRe (ROC area 0.68, *p* = 0.036) were the only predictive variables.

**Conclusions:**

In patients with NSTEAMI, we found that the novel global strain parameter of left atrial function is a valuable predictor of combined cardiovascular events over conventional echocardiography and may therefore be an important clinical tool for risk stratification in the acute phase of NSTEAMI.

## Background

Coronary artery diseases (CAD) are the most common cardiac disease, which affect more than twenty million adults in China. Acute myocardial infarction (AMI), acting as a canonical component of CAD, contributes to approximately one million identified cardiac events every year. When ST elevation of AMI patient is present, treatment is aimed at reperfusion by opening the occluded coronary artery, either by thrombolysis or by percutaneous coronary intervention (PCI). Due to presence of more sensitive biochemical markers of myocardial necrosis, an increasing number of patients with non-ST-segment-elevation acute myocardial infarction(NSTEAMI) are diagnosed [1]. These patients may develop substantial myocardial infarction (MI), but criteria for acute reperfusion therapy is rarely fulfilled [2] and clinical outcome is doubted, when compared with patients with ST-segment-elevation acute myocardial infarction.

According to previous studies, LA function index is a powerful predictor of mortality in AMI patients and provides prognostic information incremental to clinical data as well as reduced left ventricular ejection fraction (LVEF) [3]. Recently, two-dimensional speckle tracking echocardiography (STE) has been introduced as an accurate technique for quantifying regional LA function, with more comprehensive and reliable echocardiographic resolution compared with traditional methods [4]. In contrast to tissue Doppler imaging, STE shows the advantage of angle-independence, as well as being less affected by reverberations, side lobes and drop out artifacts [5]. Recently, strain echocardiography had been validated as a prognostic indicator of cardiovascular diseases [6, 7].We hypothesized that patients with NSTEAMI have impaired function of LA as well as left ventricular (LV). The aim of our study is to explore the utility of strain echocardiography in the assessment of LA function and prognostic value in patients with NSTEAMI undergoing elective PCI.

## Methods

### Patient selection

A total of 65 patients (51 males and 14 females; mean age of 60.7 ± 9.8 years) with a diagnosis of NSTEAMI confirmed at the referring hospital by elevated troponin I above the 99 % percentile and positive ECG (Evidence of ischemia was defined as any ST-deviation >0.5 mm or symmetrical T-wave inversion >3 mm in two or more contiguous leads) were enrolled from March 2012 to March 2013. All patients were considered clinically and hemodynamically stable during index admission, and none were referred for urgent coronary intervention. Time from hospital admission to elective PCI was 5.2 ± 0.8 days (range, 2 to 7 days). Patients with atrial fibrillation or flutter, valvular heart disease (of mild or greater severity), or a history of previous myocardial infarction were excluded.

All the patients had received optimized anti-ischemic (beta-blockers, nitrates), dual antiplatelet and antithrombotic therapy (aspirin, clopidogrel, enoxaparin and GP IIb/IIIa antagonists if indicated according to recent international guidelines). The study protocol was approved by the Ethics Committee of The Second Affiliated Hospital of Soochow University (Suzhou, China). Each participant provided written informed consent to be included in the study.

### Coronary angiographic procedure

Coronary angiography was performed on clinical indication by standard (Judkins) technique using digital imaging acquisition and storage. Direct stenting was allowed and various kinds of pre-dilatation devices and manual aspiration devices were allowed in case of acute myocardial infarction with severe thrombus in target artery.

### Standard echocardiography

The patients were investigated by transthoracic echocardiography immediately prior to coronary angiography and after 6 months of follow-up on a Vivid7 ultrasound system (GE, USA) equipped with a phased array transducer (frequency range of 1.7–3.4 MHz). M-mode echocardiography was used to measure LA diameter and LV end-diastolic and end-systolic diameters. LV ejection fraction (LVEF) was calculated from 4-chamber and 2-chamber view, using the modified Simpson rule. Three separate LA volumes were computed following American Society of Echocardiography guidelines using biplane modified Simpson’s method [5]. Left atrial maximum volume (LAVmax) at the end of LV systole, just before the opening of mitral valve and LA minimum volume (LAVmin) at the end of LV diastole, right after the closure of the mitral valve, were measured and LA pre-atrial volume (LAVp) was obtained from the diastolic frame before initial mitral valve reopening elicited by atrial contraction. Left atrial volume index (LAI) = left atrial maximum volume/body surface area; LA reservoir function was assessed using the following equation: LA total EF = (LAVmax-LAVmin)/LAVmax; Passive left atrial ejection fraction (LAPEF) = (LAVmax-LAVp)/LAVmax; Left atrial active ejection fraction (LAAEF) = (LAVp - LAVmin) / LAVp.

### Tissue doppler imaging echocardiography

LV diastolic function was explored by pulsed Tissue Doppler imaging, placing the sample volume at the level of mitral lateral and septal annulus from the apical four chamber view. Mean peak systolic (S), early diastolic (E’), and late diastolic (A’) annular velocities were obtained by averaging respective values measured at the septal and lateral sides of the mitral annulus. Mean Em and the derived mean E’/A’ ratio were used as load-independent markers of ventricular diastolic relaxation. Mean E/E’ ratio was also calculated.

### Two-dimensional speckle tracking echocardiography(STE)

**Fig. 1:**
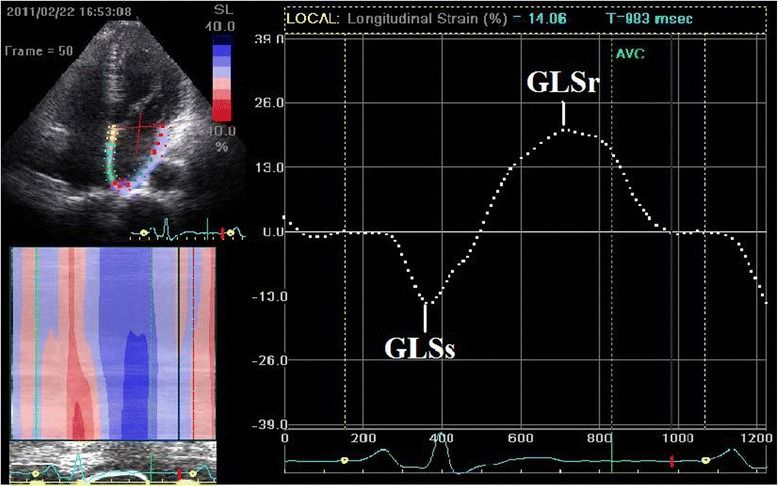
Measurement of global longitudinal left atrial strain from an apical four-chamber view. The white dashed curve represents the global longitudinal atrial strain along the cardiac cycle. GLSs = global left atrial peak negative strain during atrial systole. GLSr = global left atrial peak positive strain during ventricle systole. AVC = aortic valve closure

**Fig. 2:**
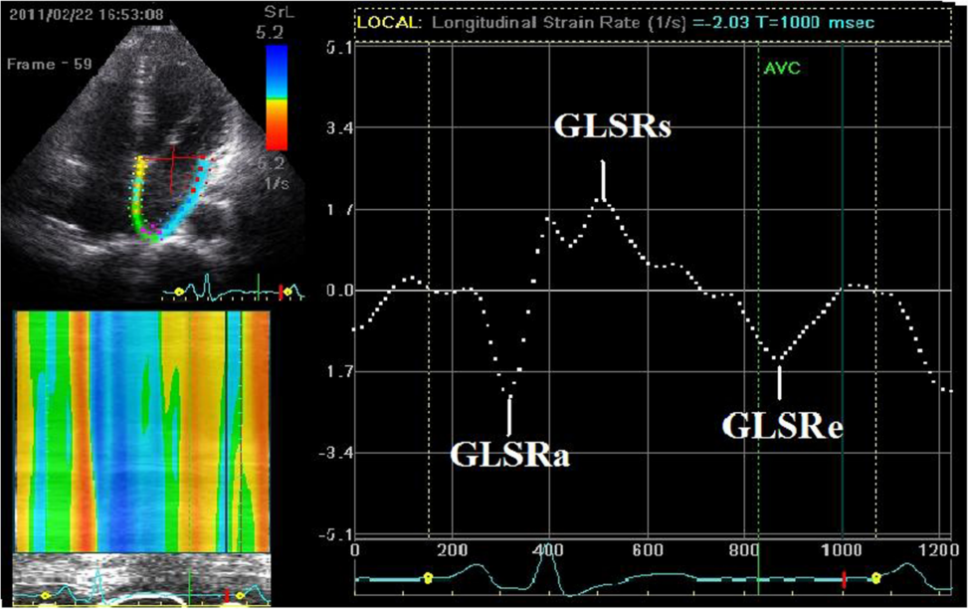
Measurement of global longitudinal left atrial strain rate from an apical four-chamber view. The white dashed curve represents the global longitudinal strain rate during the cardiac cycle. GLSRa = global left atrial peak negative strain rate during late ventricular diastole. GLSRs = global left atrial peak positive strain rate during ventricle systole. GLSRe = global left atrial peak negative strain rate during early ventricular diastole . AVC = aortic valve closure

In the setting of 2D-STE analysis, three consecutive heart cycles were recorded and averaged. The frame rate was set between 60 and 90 frames per second. Echocardiograms were digitally stored and later analyzed off-line using acoustic-tracking software (Echo-Pac version 7.0, GE Vingmed). LA endocardial surface was manually traced in each view with a point-and-click approach. An epicardial surface tracing was then automatically generated by the software, creating a region of interest (ROI). Afterwards, The ROI was divided into three segments: annular, mid and roof, whereas the resultant tracking quality for each segment is automatically scored for evaluation as either acceptable or non-acceptable. Finally, the software generated strain and strain rate curves for each atrial segment [8]. We acquired global longitudinal LA peak negative strain during atrial systole (GLSs), peak positive strain during ventricle systole (GLSr), peak positive strain rate during ventricle systole (GLSRs), peak negative strain rate during early ventricular diastole (GLSRe) and peak negative strain rate during late ventricular diastole (GLSRa) (Figs. 1 and 2), respectively [9]. Mean global longitudinal LA S/SR of three views was calculated as following: (LA S/SR in apical long axis view + LA S/SR in 4-chamber + LA S/SR in 2-chamber view)/3.We identified 65 subjects in our database. Of these patients, 14 subjects were excluded from analysis for inadequate electrocardiograms or inadequate imaging quality due to acquisition with low frame rates (*n* = 8), LA foreshortening or inadequate acoustic windows (*n* = 6). The final study population consisted of 51 individuals (43 males and 8 females; mean age of 62.9 ± 11.1 years).

### Follow-up and study endpoints

Fifty one NSTEAMI patients were followed from March 2011 to March 2013 with follow-up telephone conversations or review of medical records. Patients were monitored regularly for the occurrence of cardiac mortality and adverse cardiac events. The primary end point of this study was combined cardiac events, which were defined as combined cardiac-cause mortality and re-hospitalization due to adverse cardiac events such as exacerbation of heart failure or acute myocardial ischemia. Cardiac death was defined by clinical data of acute myocardial infarction and/or fatal cardiac arrhythmias and/or refractory congestive heart failure. The date of the last interview or review was used to calculate the follow-up duration.

### Statistical analysis

Data was analyzed using the statistical software package (SPSS, Rel 13.0, Chicago: SPSS Inc.). Continuous data were presented as mean ± SD. Differences between the baseline and 6 months groups were compared using the independent *t*-test. Categorical parameters are presented as numbers (%), and were analyzed by *χ*^2^ test. Univariable Cox proportional-hazards regression models were used to identify independent predictors of late cardiac events. The risk of a given variable was expressed by a hazard ratio (HR) with corresponding 95 % confidence intervals. Receiver-operating characteristic (ROC) curves were constructed, and areas under curves were measured to determine cutoff values with maximum sensitivity and specificity. A 2-tailed p-value < 0.05 was considered significant for statistical inference.

## Results

Over an average of 12 months (range 6 to 17 months, 12 ± 3 months) follow-up, a sum of 22 cardiovascular events(20 patients) have been recorded, including 2 patients death (9.1 %) due to a cardiac cause, 11 progressive heart failure (50.0 %), and 9 recurrent acute coronary syndrome(ACS) (40.9 %) (Table 1). According to clinical follow-up results, the overall population was divided into two groups: patients with (*n* = 20) and without (*n* = 31) cardiac events.

**Table 1:** Follow-up combined cardiovascular events

Cardiovascular Events	*n* = 22(20 patients)
Cardiovascular death	2 (9.10 %)
Re-hospitalization	20 (90.90 %)
Progressive heart failure	11 (50 %)
Recurrent ACS	9 (40.90 %)

*ACS* acute coronary syndrome

### Clinical and angiographic characteristics

Baseline clinical and angiographic characteristics of two groups are shown in Table 2. No significant differences were evidenced between the two groups, except for a higher prevalence of Hyperlipidemia and less final TIMI 3 flow in had events patients.

**Table 2:** Clinical and catheterization data of the study population of NSTEAMI patients Stratified by Event Status (*n* = 51)

Characteristics	Had events (*n* = 20)	No events (*n* = 31)	*p*
Clinical data			
Age (years)(means)	63.2 ± 9.46	60.9 ± 12.17	0.274
Male (%)	18(90.0 %)	25(80.6 %)	0.138
Female (%)	2 (10.0 %)	6 (19.4 %)	0.068
Body mass index (kg/m^2^)	1.81 ± 0.17	1.78 ± 0.21	0.454
Heart rate (beats/min)	78 ± 8.11	74 ± 10.23	0.228
Systolic blood pressure (mmHg)	144 ± 18	138 ± 12	0.152
Diastolic blood pressure (mmHg)	68 ± 19	65 ± 17	0.098
Diabetesmellitus (%)	6(30.0 %)	11(35.5 %)	0.148
Hyperlipidemia (%)	12(60.0 %)	14(45.2 %)	0.048*
Catheterization data			
Native diseased vessel (%)			
One vessel	7(35.0 %)	11(35.5 %)	0.216
Two vessels	3(15.0 %)	7(22.6 %)	0.192
Three vessels	10(50.0 %)	13(41.9 %)	0.323
Treated vessel, n (%)			
LAD	5(25.0 %)	6(19.4 %)	0.277
LCX	3(15.0 %)	5(16.1 %)	0.284
RCA	1(5.0 %)	2(6.5 %)	0.408
Final TIMI flow (%)			
3	19(95 %)	29(93.5 %)	0.054
<3	3	0	<0.001**

*LCX* left circumflex coronary artery, *LAD* left anterior descending, *RCA* right coronary artery, *TIMI* thrombolysis in myocardial infarction

*P < 0.05, **P < 0.01, between Had events group and No events group

### Echocardiography characteristics

Conventional and TDI echocardiography parameters of LA and LV function are shown in Table 3. Table 4 displays baseline STE data on three views and baseline mean electrocardiographic data, as well as 6 months mean echocardiography data for the entire study patients. Patients with cardiac events had significantly decreased LAPEF(25.16 ± 12.26 vs 29.02 ± 10.22, *p* = 0.028), baseline mean GLASRs(1.39 ± 0.48 vs 1.62 ± 0.55, *p* = 0.003), mean Glaser(−1.07 ± 0.42 vs−1.25 ± 0.48, *p* = 0.013) and 6 months mean GLASRs(1.73 ± 0.48 vs 1.98 ± 0.61, *p* = 0.017),but increased E/E’ ratio(6.9 ± 2.7 vs 5.8 ± 1.8, *p* = 0.037).

**Table 3:** Conventional and TDI electrocardiographic characteristics of patients

Characteristic	Had events (*n* = 20)	No events (*n* = 31)	*p*
LAVmax (ml)	62.18 ± 17.64	59.32 ± 11.77	0.104
LAVmin (ml)	26.56 ± 12.59	25.17 ± 9.58	0.262
LAVp (ml)	43.81 ± 15.59	43.06 ± 16.17	0.303
LAI (ml/m^2^)	35.78 ± 10.15	34.88 ± 11.51	0.089
LAPEF (%)	25.16 ± 12.26	29.02 ± 10.22	0.028*
LAAEF (%)	43.19 ± 9.67	45.33 ± 12.71	0.078
LA total EF (%)	58.06 ± 11.04	60.12 ± 10.52	0.054
LVEF (%)	56.18 ± 10.01	62.44 ± 11.92	0.089
E (cm/s)	77 ± 22	74 ± 16	0.434
A (cm/s)	82 ± 17	88 ± 11	0.118
E/A ratio	0.8 ± 0.2	0.8 ± 0.3	0.787
DT, ms	258 ± 44	230 ± 56	0.668
E’ (cm/s)	11.2 ± 2.5	12.5 ± 1.9	0.184
A’ (cm/s)	12.8 ± 2.2	11.6 ± 1.4	0.098
E/E’ ratio	6.9 ± 2.7	5.8 ± 1.8	0.037*

*TDI* Tissue Doppler Imaging, *LAVmax* Left atrial maximum volume, *LAVmin* LA minimum volume, *LAVp* LA pre-atrial volume, *LAI* Left atrial volume index, *LAPEF* left atrial passive ejection fraction, *LAAEF* Left atrial active ejection fraction, *LA total EF* left atrial total ejection fraction, *LVEF* left ventricular ejection fraction, *E*:mitral early diastolic peak velocity, *A* mitral late diastolic peak velocity, *E/A* ratio of early to late diastolic transmitral flow velocity, *DT* deceleration time, *E’* myocardial early diastolic peak velocity, *A’* myocardial late diastolic peak velocity, *E/E’ ratio* ratio of mitral to myocardial early diastolic peak velocity

*P < 0.05, between Had events group and No events group

**Table 4:** Left atrial STE characteristics of NSTEAMI patients

Characteristic	Had events (*n* = 20)	No events (*n* = 31)	*p*
Apical long axis			
GLSs (%)	−13.46 ± 7.70	−14.59 ± 7.62	0.607
GLSr (%)	13.02 ± 9.64	16.86 ± 9.31	0.163
GLSRs (s^−1^)	1.57 ± 0.58	2.04 ± 0.82	0.027*
GLSRe (s^−1^)	−1.31 ± 0.77	−1.51 ± 0.77	0.366
GLSRa (s^−1^)	−1.87 ± 1.10	−2.08 ± 0.95	0.489
Four-chamber			
GLSs (%)	−12.32 ± 5.65	−15.42 ± 7.31	0.095
GLSr (%)	11.33 ± 7.37	14.74 ± 7.62	0.116
GLSRs (s^−1^)	1.38 ± 0.79	1.56 ± 0.84	0.441
GLSRe (s^−1^)	−1.04 ± 0.47	−1.30 ± 0.59	0.084
GLSRa(s^−1^)	−1.52 ± 0.85	−1.99 ± 1.08	0.092
Two-chamber			
GLSs (%)	−11.85 ± 3.77	−13.67 ± 2.92	0.071
GLSr (%)	9.78 ± 5.63	11.30 ± 4.98	0.314
GLSRs (s^−1^)	1.21 ± 0.45	1.25 ± 0.36	0.739
GLSRe (s^−1^)	−0.87 ± 0.51	−1.03 ± 0.47	0.271
GLSRa(s^−1^)	−1.51 ± 0.62	−1.81 ± 0.48	0.057
Baseline Mean			
GLSs (%)	−12.54 ± 4.61	−14.56 ± 4.76	0.136
GLSr (%)	11.38 ± 5.10	14.30 ± 5.58	0.059
GLSRs (s^−1^)	1.39 ± 0.48	1.62 ± 0.55	0.003**
GLSRe (s^−1^)	−1.07 ± 0.42	−1.25 ± 0.48	0.013*
GLSRa(s^−1^)	−1.64 ± 0.67	−1.86 ± 0.71	0.138
6 months Mean			
GLSs (%)	−12.76 ± 4.78	−13.88 ± 5.21	0.094
GLSr (%)	13.22 ± 5.42	14.74 ± 6.89	0.228
GLSRs (s^−1^)	1.73 ± 0.48	1.98 ± 0.61	0.017*
GLSRe (s^−1^)	−1.69 ± 0.46	−1.75 ± 0.52	0.064
GLSRa(s^−1^)	−1.84 ± 0.49	−1.98 ± 0.37	0.445

*GLSs* global longitudinal left atrial peak negative strain during atrial systole, *GLSr* global longitudinal left atrial peak positive strain during ventricle systole, *GLSRs* global longitudinal left atrial peak positive strain rate during ventricle systole, *GLSRe* global longitudinal left atrial peak negative strain rate during early ventricular diastole, *GLSRa* global longitudinal left atrial peak negative strain rate during late ventricular diastole

*P < 0.05, **P < 0.01, between Had events group and No events group

### Prediction of clinical and echocardiography characteristics

Correlations of cardiac events were assessed using multivariable Cox proportional hazards models (Table 5), containing four echocardiography variables, which were significant predictors of combined cardiovascular events: LAPEF (HR = 1.05, 95 % CI 1.02 to 1.08, *p* = 0.003), LA total EF (HR = 1.02, 95%CI 0.99 to 1.05, *p* = 0.048), baseline mean GLSRs (HR = 1.27, 95 % CI 1.01 to 1.59, *p* =0.044) and baseline mean GLSRe (HR = 6.95,95 % CI 2.00 to 24.94, *p* = 0.002). Other clinical variables (age, sex, BMI, hypertension, diabetes mellitus, and hyperlipidemia), angiographic variables (native diseased vessel, natively occluded coronary artery, final TIMI flow, and ACC/AHA lesion type after PTCA), conventional electrocardiographic characteristics (LAVmax, LAVmin, LAVp, LAAEF, LAVI and LVEF) and STE variables (baseline mean GLSs, GLSr, GLSRa, 6-month mean GLSs, GLSr, GLSRa, GLSRs and GLSRe) tested were not significant predictors of diagnostic accuracy of cardiac events.

**Table 5:** Multivariable Predictors of Combined Cardiovascular Events by Cox Proportional Hazards Analysis

Variables	Hazard Ratio	95 % CI	Wald *χ* ^2^	*P* Value
Age	0.8	0.5–1.44	2.85	0.095
LAPEF (%)	1.05	1.02–1.08	8.88	0.003**
LA total EF (%)	1.02	0.99–1.05	3.89	0.048*
E/E’ ratio	0.96	0.93–0.98	12.64	0.001**
Baseline mean GLSRs (s^−1^)	1.27	1.01–1.59	4.05	0.044*
Baseline mean GLSRe (s^−1^)	6.95	2.00–24.95	9.58	0.002**

*LAPEF* left atrial passive ejection fraction, *LA total EF* left atrial total ejection fraction, *E/E’ ratio* ratio of mitral to myocardial early diastolic peak velocity, *GLSRs* global longitudinal left atrial peak positive strain rate during ventricle systole, *GLSRe* global longitudinal left atrial peak negative strain rate during early ventricular diastole

*P < 0.05, **P < 0.01

**Fig. 3:**
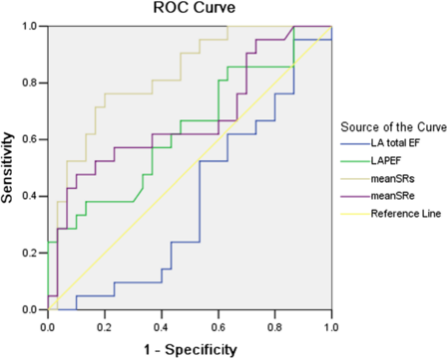
Receiver operator characteristic analyses of echocardiography parameters to predict Combined Cardiovascular Events with optimal cut-offs in patients with NSTEAMI

In accordance to the ROC analysis, baseline mean GLSRs (ROC area 0.82, *p* = 0.001) and baseline mean GLSRe (ROC area 0.68, *p* = 0.036) displayed a better prognostic value in predicting cardiac events than LAPEF (ROC area 0.64, *p* = 0.094) and LA total EF (ROC area 0.39, *p* = 0.174). The “optimal” cut off values of baseline mean GLSRs and baseline mean Glare for cardiac events were 1.62 (s^−1^) and −1.16 (s^−1^), respectively (Table 6 and Fig. 3).

**Table 6:** Receiver operating characteristics analysis of echocardiographic parameters to predict cardiovascular events

Variables	Cut off Value	ROC Area	Univariate	Se %	Sp %
		(95 % CI)	*P* Value		
LAPEF (%)	31.69	0.52(0.36–0.69)	0.781	52.4	46.7
LA total EF (%)	61.54	0.41(0.25–0.57)	0.267	46.7	43.3
E/E’ ratio	5.94	0.46(0.29–0.63)	0.605	47.6	46.7
Baseline mean GLSRs (s^−1^)	1.68	0.78(0.64–0.91)	0.001**	76.4	67.7
Baseline mean GLSRe (s^−1^)	−1.24	0.82(0.70–0.94)	0.001**	85.7	76.7

*ROC* Receiver operator characteristic, *Se* sensitivity, *Sp* specificity, *LAPEF* left atrial passive ejection fraction, *LA total EF* left atrial total ejection fraction, *GLSRs* global longitudinal left atrial peak positive strain rate during ventricle systole, *GLSRe* global longitudinal left atrial peak negative strain rate during early ventricular diastole

*P < 0.05, **P < 0.01

## Discussion

The left atrium serves as a blood reservoir during ventricular systole and a conduit for the passage of blood from the pulmonary veins into the left ventricle during early and middle ventricular diastole, as well as a booster pump increasing LV filling during late diastole [10]. Using conventional echocardiography to perform LA function analysis, three different parameters (LA total EF, LAPEF, and LAAEF) can be obtained which may latterly be used to evaluate the reservoir, conduit, and booster pump components of LA function. Chinali et al. [11] have reported that the LA ejection force has been proposed as an independent predictor of LV diastolic properties and subsequent cardiovascular events. In the present investigation, similar findings were observed. In concert with their studies, our data showed that LAPEF and LA total EF were significant predictors of cardiac events (HR = 1.05, *p* = 0.003 and HR = 1.02, *p* = 0.048, respectively) in patients with NSTEAMI after PCI.

Strain rate imaging on the basis of speckle-tracking technique represents the velocity gradient between two spatial points in relation to each other and overcomes noise artifacts associated with Doppler velocity imaging [12]. Quantification method of LA myocardial function using speckle tracking has been recently proposed [13]. In concert with conventional echocardiography, Inaba Y et al. [14] found that SRs corresponds to reservoir function and SRe corresponds to conduit function while SRa corresponds to booster pump function. Compared with S, SR seems to be less load-dependent, might be a better measure of contractility, and is theoretically more sensitive than S to myocardial pathology [15, 16].

The prognostic value of longitudinal LV strain in patients with NSTEAMI was consistent with previous reports. Park et al. [17], who studied 50 patients with acute anterior MI and primary reperfusion (PCI in 44 patients and thrombolysis in six patients) and assessed longitudinal strain by both tissue Doppler imaging (TDI) and STE in seven LV segments related to the vascular territory of the LAD artery territory. A total of 22 patients showed LV remodeling (LV dilatation with an increase in LVEDV >15 % during follow-up). Both strain assessed by TDI and assessed by speckle-tracking imaging were independent predictors of LV remodeling (odds ratio 1.430 and 1.307, respectively) and death as well as development of congestive heart failure during follow-up (odds ratio1.436 and 1.455, respectively). Recently, in a group of more than 600 patients from the Valsartan In Acute Myocardial Infarction (VALIANT) trial, Hung et al. demonstrated that both strain and strain rate (by speckle-tracking imaging) were independent predictors for death. In particular, strain rate imaging provided incremental prognostic information beyond LVEF after AMI [18]. However, there were few previous studies on the prognostic value of LA strain and strain rate in patients with NSTEAMI. In the current study, we observed the independent prognostic value of both left atrial traditional echocardiography parameters and longitudinal S and SR imaging and clinical variables in patients with NSTEAMI.As a result, multivariable Cox regression analysis showed that baseline LA Strain Rate (mean GLSRs, mean GLSRe), LAPEF and LA total EF were the independent predictor of combined cardiovascular events. Furthermore ROC analysis demonstrated that the diagnostic content of conventional echocardiography on predictor of cardiac events was less in LAPEF (ROC area 0.64, *p* = 0.094) and LA total EF (ROC area 0.39, *p* = 0.174) than STE in baseline mean GLSRs (ROC area 0.82, *p* = 0.001) and mean GLSRe (ROC area 0.68, *p* = 0.036). A cutoff value of baseline mean GLSRs 1.62 s^−1^ and mean GLSRe −1.16 s^−1^ predicted cardiac events with a sensitivity of 71.4 and 61.9 %, specificity of 83.3 and 63.3 %, respectively. Nevertheless, LAPEF and LA total EF identified cardiac events with a sensitivity of 52.4 %, 66.7 % and a specificity of 46.7 %, 53.3 %, respectively, which were lower than that of baseline mean GLSRs and mean GLSRe (Table 6 and Fig. 3). Accordingly, LA longitudinal SRs and SRe further added incremental value beyond LA ejection force in the prediction of all cause cardiovascular events. As generally accepted, LV diastolic dysfunction is a hallmark of the severity of cardiac disease, whereas the degree of diastolic impairment correlates with symptoms and prognosis more closely than LV contractive dysfunction in patients with AMI [19]. LA dysfunction represents a strong predictive marker since the atrial chamber is a window allowing comprehensive evaluation of LV diastolic dysfunction which is often difficult to analyze directly. Our study reconfirmed these findings and extended them further.

On the basis of the superiority of global SR to S and other traditional echocardiography in predicting infarct size and assessment of myocardial viability from these previous studies, we hypothesized that the average SR from all three views(long apical axis, 4-chamber and 2-chamber view) reflecting the total extent of the whole LA function damage—might provide a better prediction of clinical outcomes after MI. Our study demonstrated that both mean GLSRs and mean GLSRe improved significantly from baseline to 6-month, suggesting that LA reservoir and conduit function were restored to a certain extent in patients with NSTEAMI after PCI.

To our perspective, this study provides evidence that noninvasive provides prognostic information about the success of PCI procedure is highly desirable. With the use of this definition, mean GLSRs and mean GLSRe of left atrium, represents an optimal method with high specificity and sensitivity for the prediction of patients with NSTEAMI. In this regard, the value of 2D STE arises from its ability to directly measure atrial myocardium deformation that can be used at the bedside and provide additional information about atrial function of NSTEAMI to what can be obtained from a conventional echocardiographic analysis.

## Conclusions

2D-STE provides important insights into the mechanism and temporal sequence of left atrial dysfunction after NSTEAMI. As confirmed in our study, mean GLSRs and mean GLSRe, the new LA function parameters, which are measured by 2D-STE, exert better prognostic value in predicting cardiac events over conventional echocardiography and should be the preferred method in patients with NSTEAMI.

## Abbreviations

NSTEAMI: Non-ST-segment-elevation acute myocardial infarction
STE: Speckle tracking echocardiography

## References

[1] Roger VL, Killian JM, Weston SA, Jaffe AS, Kors J, Santrach PJ (2006). Redefinition of myocardial infarction: prospective evaluation in the community. Circulation.

[2] Bassand JP, Hamm CW, Ardissino D, Boersma E, Budaj A, Fernandez-Aviles F (2007). Guidelines for the diagnosis and treatment of non-ST-segment elevation acute coronary syndromes. Eur Heart J.

[3] Moller JE, Hillis GS, Oh JK, Seward JB, Reeder GS, Wright RS (2003). Left atrial volume: a powerful predictor of survival after acute myocardial infarction. Circulation.

[4] Wang Z, Tan H, Zhong M, Jiang G, Zhang Y, Zhang W (2008). Strain rate imaging for noninvasive functional quantification of the left atrium in hypertensive patients with paroxysmal atrial fibrillation. Cardiology.

[5] Lang RM, Bierig M, Devereux RB, Flachskampf FA, Foster E, Pellikka PA (2005). Recommendations for chamber quantification: a report from the american society of Echocardiography’s guidelines and standards committee and the chamber quantification writing group, developed in conjunction with the european association of echocardiography, a branch of the european society of cardiology. J Am Soc Echocardiogr.

[6] Cho GY, Marwick TH, Kim HS, Kim MK, Hong KS, Oh DJ (2009). Global 2-dimensional strain as a new prognosticator in patients with heart failure. J Am Coll Cardiol.

[7] Stanton T, Leano R, Marwick TH (2009). Prediction of all-cause mortality from global longitudinal speckle strain: comparison with ejection fraction and wall motion scoring. Circ Cardiovasc Imaging.

[8] Serri K, Reant P, Lafitte M, Berhouet M, Le Bouffos V, Roudaut R (2006). Global and regional myocardial function quantification by two-dimensional strain: application in hypertrophic cardiomyopathy. J Am Coll Cardiol.

[9] Kim DG, Lee KJ, Lee S, Jeong SY, Lee YS, Choi YJ (2009). Feasibility of two-dimensional global longitudinal strain and strain rate imaging for the assessment of left atrial function: a study in subjects with a low probability of cardiovascular disease and normal exercise capacity. Echocardiography.

[10] Abhayaratna WP, Seward JB, Appleton CP, Douglas PS, Oh JK, Tajik AJ (2006). Left atrial size: physiologic determinants and clinical applications. J Am Coll Cardiol.

[11] Chinali M, de Simone G, Roman MJ, Bella JN, Liu JE, Lee ET (2005). Left atrial systolic force and cardiovascular outcome. The Strong Heart Study. Am J Hypertens.

[12] Hashimoto I, Li X, Hejmadi BA, Jones M, Zetts AD, Sahn DJ (2003). Myocardial strain rate is a superior method for evaluation of left ventricular subendocardial function compared with tissue doppler imaging. J Am Coll Cardiol.

[13] D’Andrea A, Caso P, Romano S, Scarafile R, Cuomo S, Salerno G (2009). Association between left atrial myocardial function and exercise capacity in patients with either idiopathic or ischemic dilated cardiomyopathy: a two-dimensional speckle strain study. Int J Cardiol.

[14] Inaba Y, Yuda S, Kobayashi N, Hashimoto A, Uno K, Nakata T (2005). Strain rate imaging for noninvasive functional quantification of the left atrium: comparative studies in controls and patients with atrial fibrillation. J Am Soc Echocardiogr.

[15] Greenberg NL, Firstenberg MS, Castro PL, Main M, Travaglini A, Odabashian JA, Main M, Travaglini A, Odabashian JA (2002). Doppler-derived myocardial systolic strain rate is a strong index of left ventricular contractility. Circulation.

[16] Thomas JD, Popovic ZB (2006). Assessment of left ventricular function by cardiac ultrasound. J Am Coll Cardiol.

[17] Park YH, Kang SJ, Song JK, Lee EY, Song JM, Kang DH (2008). Prognostic value of longitudinal strain after primary reperfusion therapy in patients with anterior-wall acute myocardial infarction. J Am Soc Echocardiogr.

[18] Hung CH, Shin SH, Hassanein A (2008). Strain and strain rate imaging are independent predictors of mortality after high-risk myocardial infarction[abstract]. J Am Coll Cardiol.

[19] Poulsen SH, Jensen SE, Moller JE, Egstrup K (2001). Prognostic value of left ventricular diastolic function and association with heart rate variability after a first acute myocardial infarction. Heart.

